# A Prescription Digital Therapeutic to Support Unsupervised Buprenorphine Initiation for Patients With Opioid Use Disorder: Protocol for a Proof-of-Concept Study

**DOI:** 10.2196/43122

**Published:** 2023-01-20

**Authors:** Hilary Luderer, Nicole Enman, Robert Gerwien, Stephen Braun, Samantha McStocker, Xiaorui Xiong, Carrington Koebele, Christopher Cannon, Joseph Glass, Yuri Maricich

**Affiliations:** 1 Pear Therapeutics (US), Inc Boston, MA United States; 2 Whitman-Walker Institute Washington, DC United States; 3 Kaiser Permanente Washington Health Research Institute Seattle, WA United States

**Keywords:** buprenorphine, digital therapeutics, opioid use disorder, OUD, prescription digital therapeutic, PDT, reSET-O, unsupervised buprenorphine initiation, medication adherence, digital health intervention

## Abstract

**Background:**

Home-based (unsupervised) buprenorphine initiation is considered safe and effective, yet many patients report barriers to successful treatment initiation. Prescription digital therapeutics (PDTs) are software-based disease treatments regulated by the US Food and Drug Administration (FDA). The reSET-O PDT was authorized by the FDA in 2018 and delivers behavioral treatment for individuals receiving buprenorphine for opioid use disorder (OUD). A prototype PDT (PEAR-002b) designed for use with reSET-O was developed to assist in unsupervised buprenorphine initiation.

**Objective:**

The primary objective of this pilot study is to evaluate the acceptability of PEAR-002b in individuals with OUD who use it to support buprenorphine initiation, their unsupervised buprenorphine initiation success rate, and their medication adherence.

**Methods:**

Ten adults with OUD will be recruited for acceptability and feasibility testing. Outcomes will be assessed using week-1 visit attendance, participant interviews and satisfaction surveys, and urine drug screening (UDS). Three tools will be used in the study: PEAR-002b, reSET-O, and EmbracePlus. PEAR-002b includes a new set of features designed for use with reSET-O. The mechanism of action for the combined PEAR-002b and reSET-O treatment is a program of medication dosing support during week 1 of the initiation phase, cognitive behavioral therapy, and contingency management. During the medication initiation phase, participants are guided through a process to support proper medication use. PEAR-002b advises them when to take their buprenorphine based on provider inputs (eg, starting dose), self-reported substance use, and self-reported withdrawal symptoms. This study also administers the EmbracePlus device, a medical-grade smartwatch, to pilot methods for collecting physiologic data (eg, heart rate and skin conductance) and evaluate the device’s potential for use along with PDTs that are designed to improve OUD treatment initiation. Home buprenorphine initiation success will be summarized as the proportion of participants attending the post–buprenorphine initiation visit (week 1) and the proportion of participants who experience buprenorphine initiation–related adverse events (eg, precipitated withdrawal). Acceptability of PEAR-002b will be evaluated based on individual participants’ ratings of ease of use, satisfaction, perceived helpfulness, and likelihood of recommending PEAR-002b. Medication adherence will be evaluated by participant self-report data and confirmed by UDS. UDS data will be summarized as the mean of individual participants’ proportion of total urine samples testing positive for buprenorphine or norbuprenorphine over the 4-week study.

**Results:**

This project was funded in September 2019. As of September 2022, participant enrollment is ongoing.

**Conclusions:**

This is the first study to our knowledge to develop a PDT that assists with unsupervised buprenorphine initiation with the intent to better support patients and prescribers during this early phase of treatment. This pilot study will assess the acceptability and utility of a digital therapeutic to assist individuals with OUD with unsupervised buprenorphine initiation.

**Trial Registration:**

ClinicalTrials.gov NCT05412966; https://clinicaltrials.gov/ct2/show/NCT05412966

**International Registered Report Identifier (IRRID):**

PRR1-10.2196/43122

## Introduction

Unprecedented rates of opioid misuse and mortality have led to the current opioid epidemic in the United States [1], with approximately 2.5 million people in the United States meeting the criteria for opioid use disorder (OUD) in 2020 [2]. OUD is a chronic condition with numerous physical, psychological, and personal consequences, including a high risk of death, which has risen in recent years due to the increasing prevalence of potent synthetic opioids (predominantly fentanyl) [3]. According to the latest reports, 74,754 adults died from opioid-related overdoses in the 12 months ending September 2021, representing a daily average of 205 deaths [1], a 55% increase since September 2019 [4]. Opioid-related overdoses now account for 75% of fatal overdoses of all substances, up from 70.6% in 2019, with synthetic opioids accounting for 86.6% of opioid-related deaths [4].

Initiation of evidence-based medications for opioid use disorder (MOUD), such as buprenorphine, methadone, or naltrexone, remains limited; in 2020, only 11.2% of those with OUD received MOUD treatment [2]. One of the key barriers is limited access to MOUD treatment [2], which is amplified by health care provider reservations about prescribing buprenorphine for OUD. Key reasons for this hesitancy include logistical barriers, perceptions that people with addiction are difficult to treat, insufficient time and staff support, limited provider and staff knowledge and training, and lack of a support network of experienced prescribers [5-8]. Despite the recent removal of the “X waiver” training requirement, prescribers may still feel they are not adequately equipped to begin prescribing for the above reasons [9].

For individuals who do access MOUD, medication adherence is unsatisfactory, and medication discontinuation rates are high [10,11]. Buprenorphine discontinuation rates at 6 months range from 50% to 65%, with most discontinuations occurring within the first 3 months of treatment, particularly within the first 30 days, which is a crucial phase that includes initiation and titration of buprenorphine to achieve dose stabilization [10-14]. Evidence from different real-world settings suggests that MOUD discontinuation is estimated to increase the likelihood of relapse by roughly 10 times, with relapse being strongly associated with overdose and death [15-17].

The medication initiation phase for buprenorphine, in particular, is critical. Patients who have negative experiences initiating buprenorphine may not continue treatment beyond the first day of dosing [18]. Successful initiation of buprenorphine requires the patient to be in mild to moderate withdrawal before they begin the medication [19]. People’s desire to avoid experiencing withdrawal symptoms is a primary reason they continue to use and can be a major barrier to beginning treatment [20]. Buprenorphine initiation is a high-touch process for clinicians that requires educating the patient on not only the risks and benefits of buprenorphine, but how the medication works, when to start treatment, and how to manage emotions like fear and anxiety that can complicate the process.

Providing an accessible tool that supports both the patient and clinician by offering education as well as motivational and skills-based support during this important phase of treatment could enhance the patient experience and help improve clinician willingness to prescribe. Unsupervised buprenorphine initiation, whether supported via telemedicine or traditional in-person provider interactions, is considered safe and effective and may be an equitable and accessible way to increase the rate of successful MOUD initiation [19]. One potential limitation of unsupervised MOUD initiation is the inability of physicians to monitor patient biometrics (eg, pulse, blood pressure, and oxygen saturation levels), which can inform treatment and dosing decisions. New devices exist to monitor biometrics, but whether unsupervised buprenorphine initiation protocols could benefit from data collected by passive biometric monitoring is unknown.

Another technology that may potentially be leveraged to improve the MOUD initiation process is prescription digital therapeutics (PDTs), which are software-based disease treatments evaluated for safety and effectiveness in randomized controlled trials and authorized by the US Food and Drug Administration (FDA). Prescribed by treating physicians and delivered on mobile devices, PDTs can expand access to evidence-based behavioral therapies across a variety of disease states, including OUD.

The reSET-O PDT was authorized by the FDA in 2018 for patients with OUD being treated with buprenorphine. The PDT delivers two evidence-based mechanisms of action: (1) an OUD-specific form of cognitive behavioral therapy (CBT) based on the community reinforcement approach, delivered as on-demand lessons and interactive text, video, and audio exercises and (2) reinforcement and motivation for lesson completion and abstinence via contingency management (CM), which involves eligibility for positive reinforcement messages or monetary digital gift cards. Moreover, there is currently no FDA-authorized PDT that provides assistance with home initiation of MOUD. Opportunities thus exist to better support patients with OUD in the medication initiation phase and to provide strategies to lower potential barriers to the implementation of buprenorphine MOUD treatment in clinical practice.

Given the importance of buprenorphine adherence and the need to better support both patients and providers during the initiation process, we are conducting a pilot study to evaluate PEAR-002b, an investigational digital therapeutic intended to be used with reSET-O and designed to support patients during unsupervised initiation of buprenorphine. The study also includes a wearable device capable of capturing and transmitting biometric data to evaluate the feasibility of biomarker development.

## Methods

### Study Objectives

The objective of this pilot study is to evaluate device acceptability, home buprenorphine initiation success rate, and medication adherence rate among participants with OUD who use the PEAR-002b investigational tool.

### Study Design

This will be a qualitative observational cohort study with a recruitment goal of 10 adults with OUD (inclusion and exclusion criteria are listed below).

### Study Population and Sample Size

The trial will recruit 10 adults diagnosed with OUD. The investigator will ensure that participants being considered for the study meet all eligibility criteria (details are provided in the Study Procedures and Assessments section). No additional criteria will be applied by the investigator to ensure the study population will be representative of all eligible participants. Participants may undergo rescreening with approval of the medical monitor. Due to the exploratory nature of the study, no formal sample size calculations are required.

### Inclusion Criteria

Study inclusion criteria are as follows: written informed consent provided prior to the performance of any study-specific assessments; age ≥18 years; English proficiency sufficient to meaningfully participate in the consent process, assessment, and intervention; a *Diagnostic and Statistical Manual of Mental Disorders, Fifth Edition* (*DSM-5*) diagnosis of OUD made by a treating clinician; desire to start buprenorphine treatment; capability of using common software applications on an internet-enabled mobile device (smartphone or tablet); interest in testing or using PEAR-002b; no prior history of reSET-O use; no participation in investigational drug trials within the past 30 days (or within 5 half-lives of any study drug, whichever is longer) of enrollment; and a judgment that the subject is appropriate for participation by the treating clinician.

### Exclusion Criteria

Study exclusion criteria are as follows: currently on methadone or naltrexone pharmacotherapy; currently on buprenorphine MOUD treatment; planning to move out of the geographic area within 2 months; insufficient English fluency to participate in the consent process, intervention, or assessment; inability to comply with study procedures due to physical or mental condition; currently receiving inpatient treatment for OUD; and pregnancy.

### Study Setting and Recruitment

The study will be conducted at a single site in the United States. Participants will be recruited verbally by participating clinicians and via community advertising.

### Study Tools

#### Overview

Three tools will be used in the study: PEAR-002b, reSET-O, and EmbracePlus. PEAR-002b is a standalone software app that includes a new set of features designed for use with the reSET-O PDT. The mechanism of action for the combined PEAR-002b and reSET-O treatment is a program of medication dosing support during week 1 of the buprenorphine initiation phase, including psychoeducation, CBT, and CM. During the first week of treatment (ie, the buprenorphine initiation phase) participants are guided through the medication initiation process with psychoeducation to support proper medication use and a self-report withdrawal scale to advise the participant on when to take their buprenorphine. During the use of reSET-O, which occurs in weeks 2 to 4, participants are instructed to complete 4 lessons per week. The EmbracePlus device is a medical-grade smartwatch that will be used to collect physiologic data (eg, heart rate and skin conductance) from participants who agree to use the device.

The smartphones loaned to participants in this study include an installed security feature that blocks downloads of other apps, potentially limiting their market value and thus reducing the risk that the phones will not be returned at the conclusion of the study.

#### PEAR-002b Summary Description

PEAR-002b includes an algorithm designed to support buprenorphine dosing and provide associated therapeutic content related to medication and recovery. Medication dosing recommendations are based on provider input (eg, starting dose) and reported recent substance use. A withdrawal symptom scale based on the Subjective Opiate Withdrawal Scale helps participants identify when to take their medication and how to titrate their medication dose to manage withdrawal symptoms. Development of all features and content was informed by feedback generated through participant interviews and prototype testing sessions.

The PEAR-002b content was designed to maximize the efficacy of buprenorphine treatment by educating participants on the importance of proper buprenorphine use and adherence in recovery. Videos and text-based modules address the following topics: preparing for buprenorphine initiation; recognizing, coping with, and managing common opioid withdrawal symptoms prior to buprenorphine initiation; proper initiation of buprenorphine; techniques for sublingual or buccal dosing of buprenorphine to maximize absorption and therapeutic drug levels; how medication adherence supports treatment goals; misconceptions and stigma pertaining to buprenorphine treatment; proper storage and security of buprenorphine to minimize the risk of diversion; and the mechanism of action of buprenorphine treatment.

#### reSET-O Summary Description

PEAR-002b is designed as an optional feature of the reSET-O PDT, which provides an 84-day course of behavioral treatment for OUD as an adjunct to standard buprenorphine MOUD treatment. The reSET-O PDT delivers behavioral therapy modeled on the Community Reinforcement Approach [21], which is a specific form of CBT designed for patients with OUD [22-24]. The PDT provides therapy through a series of 67 interactive lessons delivered via a patient-facing smartphone app. It also has a web-based dashboard for use by clinicians. A typical therapy lesson consists of a CBT component and skill-building exercises. Therapy lesson content is delivered primarily via written text but may also include videos, animations, and graphics. It also has an optional function that patients can use to set medication reminders. The first time a patient opens the app each day, they are asked to report whether they have taken their buprenorphine. Patients who report not taking it are asked to indicate the reasons for nonuse.

Therapy lessons are divided into “core” and “keep learning” lessons. Core lessons cover key CBT concepts, whereas “keep learning” lessons include more in-depth information on key CBT topics covered in the core lessons, as well as additional topics that may be of interest to patients. Once a core lesson is completed, patients can unlock the next lesson. “Keep learning” lessons can be completed in any order and are accessible once the core lessons are completed.

#### EmbracePlus Summary Description

EmbracePlus is a device worn on the wrist that collects, processes, stores, and wirelessly transmits physiologic parameters to a companion app and monitoring platform. The device consists of an electronic display, display case, wristband, and sensors. The device produces 2 types of data: raw data (high-frequency data recorded by EmbracePlus sensors) and digital biomarkers (computed from raw data using algorithms). Biomarkers aggregated by EmbracePlus (1 value per minute) include pulse rate, pulse rate variability, respiratory rate, blood oxygen saturation, sleep periods, movement, skin conductance, temperature, and wearing time. In addition to collecting physiologic data via its sensors, EmbracePlus enables the user to tag specific events by pressing a button on the side of the device. Data collected via EmbracePlus flow through the Empatica Care app (a participant-facing app that notifies the participant if action is required and provides a real-time display of digital biomarkers) to Empatica Cloud (for data storage) and the Empatica Care Portal (a web app to manage study participants and monitor digital biomarkers computed by EmbracePlus).

### Ethical Considerations

#### Conduct of the Study

This study will be conducted in full accordance with all applicable policies and procedures and all applicable US federal and state laws and regulations, including the 45 Code of Federal Regulations 46, and the Health Insurance Portability and Accountability (HIPAA) Privacy Rule. Any episode of noncompliance will be documented. The investigator will perform the study in accordance with this protocol and will report unexpected problems in accordance with institutional review board (Advarra IRB Services; approval number Pro00037072) procedures and all federal requirements. Collection, recording, and reporting of data will be accurate and will ensure the privacy, health, and welfare of research participants during and after the study. All participants will be required to sign an informed consent document at the screening visit, prior to the start of any study-specific procedures. The informed consent document will include an overview of the study, including a clear description of all study procedures, potential risks and benefits of participating in the study, and alternatives to participating in the study. Participants have the right to withdraw from the study at any time and for any reason, and all participants are made aware that withdrawal will not affect their routine care.

#### Confidentiality

The anonymity of participants will be maintained. Participants will be identified by their initials and an assigned study number on electronic case report forms (e-CRFs) and other documents submitted to the study monitor. No protected health information will be stored outside of the study clinic, PEAR-002b system, reSET-O, or the encrypted HIPAA-compliant electronic data capture platform. Data collected by the PEAR-002b system will be securely transferred using industry standard encryption to the PEAR-002b backend services, which use a cloud-based infrastructure that serves and communicates with the patient-facing mobile app.

The backend services contain all data and analytics for reSET-O, PEAR-002b, and its clients (participants and clinicians). All data stored by the study devices are hosted and stored by Amazon Web Services (AWS). AWS follows a variety of internationally recognized security standards, such as the National Institute of Standards and Technology SP800-53 and HIPAA. All participant information is automatically encrypted by AWS when it is entered into the system, allowing for secure data transfer (from participant device to clinician device) and storage.

The EmbracePlus and Care apps are compliant with federal regulations, directives, and international standards for medical devices. Empatica Cloud is the infrastructure where the data recorded from EmbracePlus is collected and made accessible via web interfaces and data repositories. Empatica Cloud is HIPAA compliant. Participant data collected via EmbracePlus is securely stored and encrypted within the AWS cloud service.

Participants will be compensated for attending study visits and completing study assessments as follows (all values in US $): baseline clinic visit, $50; weekly study visit (n=4), $20; urine sample (n=5), $10; qualitative interview (n=2), $50; wearable use, $10 per study visit (n=4). Participants also have the chance to receive monetary rewards in the form of digital gift cards, ranging in value from $5 to $100, earned via the use of the CM functionality of the reSET-O study device.

### Study Procedures and Assessments

Prospective participants referred to the study will be screened for eligibility criteria by a research assistant based on the study inclusion and exclusion criteria and clinical documentation of assessments as needed. Diagnoses of OUD will be made by treating clinicians following standard clinical protocols at the study site, based on *DSM-5* criteria. Recruited participants will be given standard screenings in accordance with the assessment schedule. For the convenience of participants, screening and baseline visits may be combined. All participants will be required to sign an informed consent form at the screening visit, prior to the start of any study procedures. Participants meeting eligibility criteria will be assigned a subject identification number during the baseline visit. Study participants will continue to receive standard treatment for OUD following prescriber-recommended instructions for medication formulation and starting dose, as well as clinic visits unrelated to the study (eg, for medication management or counseling). Standard treatment will be supplemented with the therapeutic tools (PEAR-002b and reSET-O).

Participants will be enrolled for 4 weeks to assess the acceptability and ease of use of the PDT. At baseline, if participants still meet all inclusion and exclusion criteria, study staff will provide them with a phone preloaded with the study apps. Participants will be asked if they also agree to wear the EmbracePlus smartwatch for the duration of the study, and those who agree will be provided the device. Study staff will train willing participants in use of the study apps and the EmbracePlus device. Additionally, a UDS will be performed, and the Clinical Opiate Withdrawal Scale (COWS) will be used. One week later, the participant will return to the clinic and complete a survey and an interview. Study staff will conduct another UDS, administer the COWS, and ask about any adverse events (AEs) or side effects. Following this visit, the participant will return weekly for a UDS and AE assessment. At week 4, the participant will return for a UDS and complete a final survey and exit interview. Primary, secondary, and exploratory objectives and end points are outlined in Textbox 1.

**Study objectives and end points.**

**Primary objectives**

- To assess the effect of PEAR-002b on home buprenorphine initiation success rates (ie, the proportion of participants attending the post–buprenorphine initiation visit)
- To evaluate the acceptability of PEAR-002b
- To assess the effect of PEAR-002b on medication adherence rates

**Primary end points**

- Proportion of participants attending the post–buprenorphine initiation visit and the proportion of participants who experience buprenorphine initiation–related adverse events (eg, precipitated withdrawal)
- Participants’ ratings of PEAR-002b for ease of use, satisfaction, perceived helpfulness, and the likelihood they would recommend it
- Participant self-report data and confirmation by urine drug screens

**Secondary objectives**

- To assess dosing and engagement of participants with PEAR-002b
- To evaluate the contingency management reward structure

**Secondary end points**

- Participants’ use patterns of PEAR-002b, including lesson completion, self-report assessment completion, voluntary use of self-report features, and response to notifications
- Participant interviews and user satisfaction surveys

**Exploratory objectives**

- To evaluate the preliminary efficacy of PEAR-002b
- To evaluate compliance with wearing the EmbracePlus device
- To evaluate the feasibility of using digital biomarkers to predict withdrawal, craving, and opioid use

**Exploratory end points**

- Abstinence from illicit opioids and medication adherence, measured by urine drug screens, and retention in treatment, measured by time to dropout (if applicable)
- Device wearing time (eg, total hours worn and average percentage of time worn per day) measured by the device
- Prediction power based on physiologic data (eg, heart rate and skin conductance) collected via the device

### Demographics

Demographic information to be collected includes age at time of consent, sex, gender, race and predominant ethnicity, level of education, marital status, employment status, income, number of children, health insurance status, involvement in the criminal justice system, distance of residence from treatment site (in miles), and usual method of transportation.

### Medical and Medication History

Relevant medical history and current medical conditions will be collected, including current or prior treatments received for mental health disorders, hepatitis C, HIV, and chronic pain syndromes. History of substance use will be collected for the following categories: opioids, cocaine, stimulants other than cocaine, alcohol, marijuana, benzodiazepines, and other substances. Data to be collected regarding each substance include onset of use, number of years used regularly, amount used, type used (eg, pill vs powder), route of use, history of overdose, and longest period of abstinence. History of substance use treatment will also be collected, including present and prior treatment, type of treatment facility, number of treatment episodes, and current recovery activities. Nicotine use history will also be collected, including type, route, quantity, duration of use, and prior use. Medication history will include the name, dose, frequency, start and stop dates of the medication, and the indication for use. Prior experience with medication for substance use disorder/OUD will also be recorded.

### Evaluation of Phone Use

Study staff will evaluate participants’ comfort level with using smartphones and navigating through common apps.

### Interviews and Surveys

Interviews with participants will be conducted by study staff to evaluate their experiences with the PDT, particularly its ease of use and acceptability. Interviews may be performed in person or remotely via video or phone. These sessions may be recorded for transcript generation if informed consent is obtained from the participant, investigator, or both. Participant surveys will be completed by participants per the assessment schedule. Table 1 shows a schedule of study assessments.

**Table 1:** Schedule of study assessments.

Procedure/measure	Baseline	Post–buprenorphine initiation	Maintenance		End of study
Timepoint	Week 0 (day 0)	Week 1 (day 7, plus or minus 2 days)	Week 2 (day 14, plus or minus 2 days)	Week 3 (day 21, plus or minus 2 days)	Week 4 (day 28, plus or minus 7 days)
Informed consent	✓				
Inclusion/exclusion	✓				
Mobile use evaluation	✓				
Demographics	✓				
Medical history	✓				
Medication list/history	✓				
EmbracePlus monitor recording	✓	✓	✓	✓	✓
Urine drug screen	✓	✓	✓	✓	✓
Adverse events and side effects assessments		✓	✓	✓	✓
Clinical Opiate Withdrawal Scale	✓	✓			✓
Participant survey		✓			✓
Participant interview		✓			✓
Medication list changes		✓	✓	✓	✓
Timeline Followback Assessment	✓	✓	✓	✓	
Protocol deviations	✓	✓	✓	✓	✓

### Data Management, Study Oversight, and Monitoring

Designated staff from the study sponsor (the study monitor) will review the e-CRFs entered by study staff for completeness and accuracy. Authorized study staff will respond to queries and make any necessary changes to the data. Oversight of data management, including data collection, storage, and export; security; tracking; data analysis; and quality assurance will be the responsibility of the sponsor. The study investigator will ensure the accuracy, completeness, and timeliness of the data reported to the sponsor and be responsible for ensuring that all study staff adhere to human subjects/IRB guidelines related to data management. Data files will be backed up at regular intervals and will be accessible only by trained study staff members.

The study monitor will ensure that the research is conducted and documented according to the protocol and in accordance with the guidelines for Good Clinical Practice. Virtual study monitoring visits will be conducted at appropriate times during the study per the monitoring plan.

### AE Reporting

The investigator or designee and research site staff are responsible for the detection, documentation, classification, reporting, and follow-up of events meeting the definition of an AE or serious AE (SAE). Spontaneously reported or observed AEs will be recorded throughout the study beginning at the time the participant gives informed consent. AEs will be elicited using a nonleading question at designated time points. Regardless of seriousness, intensity, or presumed relationship to PEAR-002b, all AEs will be recorded in the source documentation from the time of consent until the end of the last study visit. AEs that occur after screening and prior to implementation of PEAR-002b will be recorded in the source documentation under medical history. AEs occurring after baseline and until the last study visit, known as treatment-emergent adverse events (TEAEs), will be recorded in the AE page of the e-CRFs. All measures required for management of TEAEs will be recorded in the source documentation. The investigator will be responsible for informing the IRB and regulatory authorities of SAEs, as required by local and regional regulations.

### Statistical Analysis Methods

This feasibility study is largely exploratory, with several end points focused on measuring participant satisfaction, attitudes, and use of the mobile app component of reSET-O and PEAR-002b. Therefore, many of the statistical analyses will be descriptive and will involve computing and reporting measures of central tendency (ie, mean and median) and variability (ie, variance and standard deviation) for the variables of interest. These statistics will highlight which aspects of the therapeutic and the experience are helpful and engaging to participants, and which aspects are less engaging.

#### Primary End Points

Home buprenorphine initiation success will be summarized as the proportion of participants attending the post–buprenorphine initiation visit (week 1) and the proportion of participants who experience buprenorphine initiation–related AEs (eg, precipitated withdrawal). Acceptability of PEAR-002b will be evaluated based on individual participants’ ratings of ease of use, satisfaction, perceived helpfulness, and likelihood of recommending PEAR-002b. Descriptive statistics will be performed on Likert-scale and multiple-choice items that assess user satisfaction and attitudes about the app. Analyses will be conducted on data collected at each assessment point throughout the study, ratings over the course of the study, and for overall user satisfaction as measured at the end of the study. Medication adherence will be evaluated by participant self-report data and confirmed by UDS (ie, a positive result for buprenorphine and norbuprenorphine). UDS data will be summarized as means of individual participants’ proportion of total urine samples testing positive for buprenorphine or norbuprenorphine over the 4-week study.

#### Secondary End Points

For dosing and engagement, descriptive statistics will be calculated for PEAR-002b module completion, assessment completion (both in the app and clinician delivered), voluntary use of in-app self-report features, and response to notifications. For CM rewards, descriptive statistics will be calculated on Likert-scale and multiple-choice items that assess participant satisfaction with and ease of use of the CM system and rewards. Descriptive analyses will examine the frequency of rewards earned throughout the study. Text analysis of qualitative responses and transcripts from interviews and focus groups will be used to identify themes in participant feedback.

#### Exploratory End Points

Abstinence will be defined by participant self-reports and confirmed by the absence of opioids (other than buprenorphine) in the UDS. UDS data will be summarized as means of individual participants’ proportion of total urine samples testing negative for opioids other than buprenorphine over the 4-week study. Treatment retention will be measured as time to drop-out (defined as the last face-to-face contact with a participant) and analyzed using the Kaplan–Meier method.

Feasibility and acceptability of EmbracePlus will be assessed by measuring the total hours and percentage of weeks wearing the device. Week-by-week averages and the total number of hours across all weeks will be assessed. Acceptability will be evaluated based on participants’ ratings of ease of use and satisfaction with EmbracePlus. Descriptive statistics will be calculated for Likert-scale and multiple-choice items that assess user satisfaction and attitudes about EmbracePlus. If physiologic data are sufficient, multiple statistical learning models will be used to evaluate if these models have sufficient power to predict withdrawal or opioid use events in advance, levels of cravings, or both.

## Results

Recruitment for this study is currently open and will continue until the projected sample size is met. A sample size of 10 participants is standard for a feasibility study.

## Discussion

PEAR-002b is being evaluated for its potential to support participants during unsupervised buprenorphine initiation. A comprehensive analysis of data collected in the first week will be used to evaluate feasibility and acceptability. Success will be demonstrated not only by attendance at the week-1 visit, but by evidence that participants engaged with PEAR-002b during the medication initiation process and found it acceptable (ie, helpful and easy to use). If the study is successful, future efforts may focus on refinement of PEAR-002b and its integration with reSET-O, in addition to the development of tools to support provider monitoring of the patient during home medication initiation. These future efforts may help mitigate inequities that impact how individuals access and engage with treatment. These tools may offer standardization and ease of implementation, ensuring proper clinical-care delivery while lightening the burden on health care workers. Future directions could also include providing patients preparing or planning unsupervised buprenorphine initiation with a list of provider-recommended over-the-counter medications to mitigate some of the withdrawal side effects, thereby encouraging patient buy-in and ownership of their health care during a process that is often perceived as uncontrollable.

Data acquired in this pilot study may also inform larger studies to evaluate the safety and effectiveness of PEAR-002b plus reSET-O. Together, PEAR-002b and reSET-O may improve the success rate of unsupervised buprenorphine initiation and medication adherence rates, which are crucial to an individual’s success in OUD treatment.

The study may also guide efforts to refine the use of biometric outputs, such as heart rate and skin conductance, to monitor physiological symptoms of withdrawal and to provide objective data that could be incorporated into the dosing algorithm of the digital therapeutic to more accurately support dose titration. Biometric data could also be included in a dashboard, which would enable clinicians to better monitor their patients. Biometrics may help alert clinicians to patients at risk of precipitated withdrawal.

This is an early feasibility study, and as such, findings may not be generalizable due to the small sample size and the lack of a comparator arm; both of these limitations will be addressed in a full-scale study, should that be pursued. The simplicity of PEAR-002b is also a limitation of the study; for instance, it does not support all sublingual buprenorphine formulations (eg, Zubsolv), nor does it support all buprenorphine initiation protocols, as the current version of PEAR-002b is based on conventional buprenorphine initiation protocols. Due to the prevalence of potent synthetic opioids such as fentanyl in the illicit drug supply, conventional buprenorphine initiation protocols have become less predictable, with an increased risk of precipitated withdrawal, necessitating the development of alternative methods, such as low-dose buprenorphine initiation [25]. In order to create a more flexible and potentially scalable digital therapeutic, future development efforts may focus on incorporating a broader range of buprenorphine initiation protocols, including low-dose initiation, along with a dashboard that enables the prescriber to select the type of protocol, buprenorphine formulation, and starting dose.

## Abbreviations

AE: adverse events
AWS: Amazon Web Services
CBT: cognitive behavioral therapy
CM: contingency management
COWS: Clinical Opiate Withdrawal Scale
DSM-5: Diagnostic and Statistical Manual of Mental Disorders, Fifth Edition
e-CRF: electronic case report form
FDA: Food and Drug Administration
HIPAA: Health Insurance Portability and Accountability
IRB: institutional review board
MOUD: medication for opioid use disorder
OUD: opioid use disorder
PDT: prescription digital therapeutic
SAE: serious adverse event
TEAE: treatment-emergent adverse event
UDS: urine drug screen

## Data Availability

Requests for data required to support the protocol will be evaluated by the sponsor and provided if deemed appropriate. All study-related information will be regarded as confidential.
